# Tumor microenvironment characterization in triple-negative breast cancer identifies prognostic gene signature

**DOI:** 10.18632/aging.202478

**Published:** 2021-02-01

**Authors:** Yan Qin, Jiehua Deng, Lihua Zhang, Jiaxing Yuan, Huawei Yang, Qiuyun Li

**Affiliations:** 1 Department of Breast Surgery, Guangxi Medical University Cancer Hospital, Nanning 530021, Guangxi, People's Republic of China

**Keywords:** tumor microenvironment, triple-negative breast cancer, immune checkpoint

## Abstract

We aimed to elucidate the landscape of tumor microenvironment (TME) in triple-negative breast cancer (TNBC). Cohorts from Gene Expression Omnibus database (N = 107) and METABRIC (N = 299) were used as the training set and validation set, respectively. TME was evaluated via single-sample gene set enrichment analysis, and unsupervised clustering was used for cluster identification. Consequently, TNBC was classified into two distinct TME clusters (Cluster 1 and Cluster 2) according to predefined immune-related terms. Cluster 1 was characterized by low immune infiltration with poor prognosis; whereas, Cluster 2 was characterized by high immune infiltration with better survival probability. Further, Cluster 1 had larger tumor volumes, while Cluster 2 had smaller tumor volumes. Finally, a TME signature for prognosis stratification in TNBC was developed and validated. In summary, we comprehensively evaluated the TME of TNBC and constructed a TME signature that correlated with prognosis. Our results provide new insights for the immunotherapy of TNBC.

## INTRODUCTION

Breast cancer (BC) is the most common cancer in women worldwide [1]. Triple-negative breast cancer (TNBC) is a subtype of BC that is negative for estrogen receptor, progesterone receptor, and human epidermal growth factor receptor-2 [2]. TNBC accounts for 15–20 % of all BC cases; however, TNBC shows stronger aggressiveness, higher degree of malignancy, and poorer prognosis than other subtypes [3, 4]. The conventional treatment modalities of TNBC include local treatment, surgery [5], systemic treatment [6], and chemotherapy [7]. However, surgical treatment is associated with a high recurrence rate, and mainly targets localized lesions. Systemic treatments (endocrine and traditional targeted treatments), which show good prognosis in BC patients with positive hormone receptors, are ineffective in TNBC. Further, chemotherapy, which is an exclusive effective treatment for patients with TNBC, has drug toxicity that may be too severe for some patients to tolerate [8]. In addition, once chemotherapeutic drug-resistance occurs, the tumors recur rapidly [9]. To prevent drug resistance, neoadjuvant chemotherapy has been utilized for TNBC treatment using a combination chemotherapy of taxane and anthracycline [10]; unfortunately, anthracycline drugs show an irreversible toxicity to the heart. Thus, a safer and more effective treatment strategy should be developed for TNBC.

Recent evidence has highlighted the important role of tumor microenvironment (TME) in cancer initiation, progression, metastasis, and therapeutic resistance [11]. The immune TME is where immune surveillance and immune escape confront each other in the context of the human immune system and tumor cells [12, 13]. Compared with other subtypes of BC, TNBC has a unique immune microenvironment with higher expression of vascular endothelial growth factor, other molecules that promote the growth and migration of tumor cells, tumor-infiltrating lymphocytes, and tumor associated macrophages [14]. Moreover, the interaction between death receptor 1 (PD-1) on T cells and programmed cell death ligand 1 (PD-L1) on tumor cells suppresses the immune system, resulting in tumor cell immune escape [15]. In fact, PD-1/PD-L1 has been an effective target for cancer immunotherapy. Therefore, to better understand the dynamics and pathogenic role of various immune cells in TNBC, it is essential to develop more effective biomarkers for the treatment of TNBC.

In this study, we analyzed the TME landscape of TNBC, performed differential expression analysis and pathway enrichment analysis to reveal the underlying molecular mechanisms, developed a TME signature, and evaluated the correlation between the TME signature and TME cells.

## RESULTS

### Different TME clusters of TNBC

**Figure 1:**
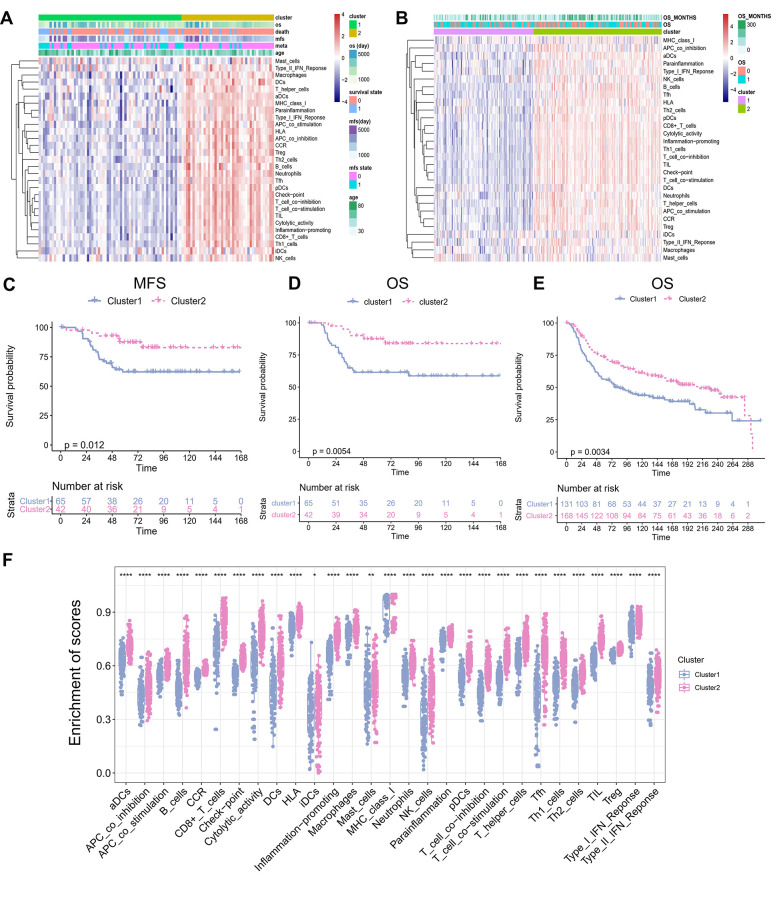
**TME Clusters of Triple-negative breast cancer.** (**A**) TNBC TME clusters in training set. Unsupervised clustering of tumor microenvironment immune cells for 107 TNBC patients from training set. Clinicopathological information including age, MFS, OS, as well as TME clusters, is shown in annotations above. (**B**) TNBC TME clustering in the validation set. Unsupervised clustering of tumor microenvironmental immune cells from 299 cases of TNBC from validation set. The OS and the number of months of survival are shown in the comments above. (**C**) Kaplan–Meier curves for Metastasis-free survival (MFS) stratified by TME clusters in the GEO cohort. (**D**) Kaplan–Meier curves for overall survival (OS) stratified by TME clusters in the training set. Hierarchical clustering was performed using Euclidean distance and ward linkage. (**E**) Kaplan–Meier curves for overall survival (OS) stratified by TME clusters in the validation set. (**F**) The abundance of immune-related terms in the Cluster 1 and 2.

Immune cell populations modulate different immune responses through the infiltration of TNBC TME and lead to antitumor effects. To investigate whether there was immune-related TME heterogeneity in TNBC, we collected 29 immune-related terms from published reports. Then, single-sample Gene Set Enrichment Analysis (ssGSEA) method was applied to assess the enrichment status of these 29 immune-related terms for each TNBC patient from the GSE58812 cohort (training set consisting of 107 TNBC samples). Unsupervised clustering of the enrichment scores of the 29 immune-related terms identified two clusters in the TNBC patients (Figure 1A): Cluster 1, which included 65 cases of TNBC, was defined as a “low immune infiltration” cluster because of its low enrichment in the immune-related terms; whereas Cluster 2, which included 42 cases of TNBC, was defined as a “high immune infiltration” cluster because of its high enrichment in the immune-related terms. This result reflected different patterns of infiltration of the immune cells based on the adaptive and innate immune systems of each patient. To validate the results of the TME clusters, we used two independent algorithms, TIMER2.0 and CIBERSORTx, to estimate the TME cell abundance. We observed similar results as most of the tumor infiltrating immune cells were significantly increased in Cluster 2 than in Cluster 1 (TIMER2.0: Supplementary Figure 1; CIBERSORTx: Supplementary Figure 2). These results indicated that Cluster 2 was associated with high immune infiltration. Further analysis showed that the patients in the two clusters manifested different outcomes. For instance, the patients in Cluster 2 displayed better metastasis-free survival (MFS; Figure 1C; P = 0.012) and overall survival (OS; Figure 1D; P = 0.0054) compared with the patients in Cluster 1. To validate the identified TME clusters, we independently performed ssGSEA and unsupervised clustering for the validation METABRIC cohort (validation set consisting of 299 TNBC patients). The results showed that all the patients were also categorized into two heterogeneous clusters with 131 and 168 patients each (Figure 1B). Significant differences in OS and pattern of cellular infiltration were observed between these two TME clusters as well (P = 0.0034; Figure 1E). Further, in Cluster 1, microenvironmental cell permeability was relatively low. In contrast, Cluster 2 exhibited both innate and adaptive immune responses with high abundance of plasmacytoid dendritic cells (pDCs), immature dendritic cells (iDCs), macrophages, B cells, and a high penetration of T cells, cytotoxic cells. We systematically compared the abundance of immune-related terms in the two clusters. The results likewise confirmed that the abundance of immune-related terms in Cluster 2 was significantly greater than in Cluster 1 (Figure 1F).

### Clinical features of different TME clusters of TNBC

We compared the clinical characteristics, including age, Nottingham prognostic index, cellularity, chemotherapy, laterality, tumor grade, tumor size, tumor stage, and positive lymph nodes between the two distinct TME clusters (Table 1). Cluster 1 (131 patients) was predominantly associated with low-immune infiltration and Cluster 2 (168 patients) was characterized by high immune infiltration. Cluster 2 had significantly smaller tumor sizes, while Cluster 1 had larger tumor sizes (P<0.001). However, the differences were not significant with respect to age, tumor grade, and tumor stage (age, P = 0.359; tumor grade, P = 0.725; tumor stage, P = 0.773), indicating that the TME clusters were independent of age, and tumor grade and stage.

**Table 1:** Clinical characteristics of the TNBC patients in the different TME clusters.

**Characteristics**	**Cluster1(%) (N=131)**	**Cluster2(%)(N=168)**	**P value**
Age (mean (SD))	56.47 (14.43)	55.00 (13.17)	0.359
NPI (mean (SD))	4.48 (0.99)	4.67 (0.91)	0.093
Cellularity (%)			
High	74 (56.5)	90 (53.6)	
Low	15 (11.5)	21 (12.5)	
Moderate	38 (29.0)	52 (31.0)	
NA	4 (3.1)	5 (3.0)	
Chemotherapy (%)			0.594
Yes	66 (50.4)	91 (54.2)	
No	65 (49.6)	77 (45.8)	
Inferred Menopausal State (%)			0.926
Pre	46 (35.1)	61 (36.3)	
Post	85 (64.9)	107 (63.7)	
Laterality (%)			0.623
Left	57 (43.5)	82 (48.8)	
Right	65 (49.6)	77 (45.8)	
NA	9 (6.9)	9 (5.4)	
Grade (%)			0.725
1	2 (1.5)	1 (0.6)	
2	16 (12.2)	20 (11.9)	
3	111 (84.7)	146 (86.9)	
NA	2 (1.5)	1 (0.6)	
Tumor size (mean (SD))	31.30 (23.41)	24.91 (12.22)	0.003
Tumor stage (%)			0.773
1	28 (21.4)	34 (20.2)	
2	59 (45.0)	71 (42.3)	
3	12 (9.2)	13 (7.7)	
NA	32 (24.4)	50 (29.8)	
Positive lymph nodes (%)			0.798
>5	18 (13.7)	26 (15.5)	
≤5	113 (86.3)	142 (84.5)	

NPI: Nottingham prognostic index.

### Differential gene and pathway analysis of different TME clusters of TNBC

**Figure 2:**
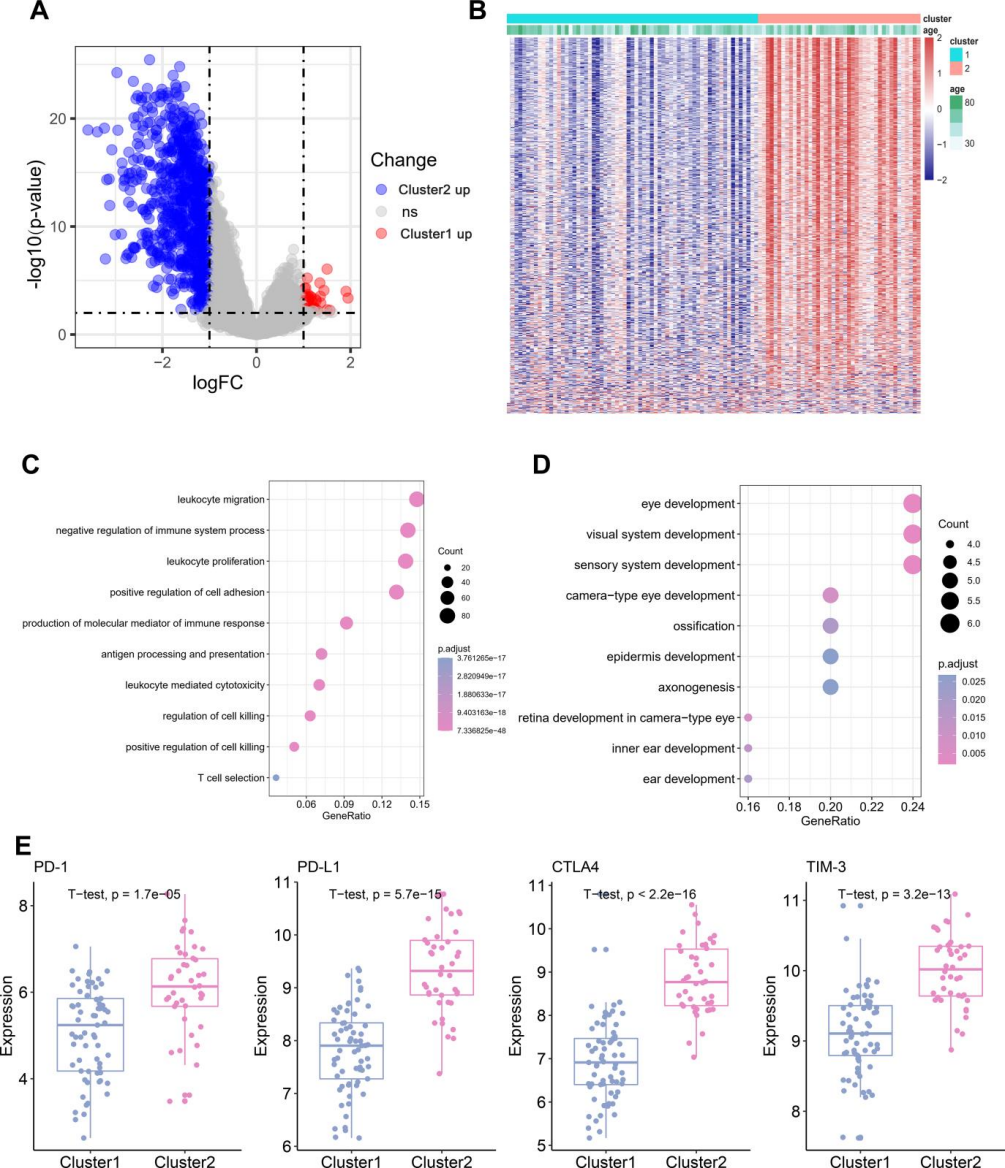
**Differential gene and pathway Analysis of Different TME Cluster of TNBC.** (**A**) The volcano plot showing the differentially expressed gene (DEGs) between TME Cluster 1 and Cluster 2. Blue is highly expressed in Cluster 2 and red is highly expressed in Cluster1. (**B**) Heatmap shows the expression of DEGs between the two clusters. (**C**) KEGG functional enrichment analyses of the up-regulated genes in Cluster 2. (**D**) KEGG functional enrichment analyses of the up-regulated genes in Cluster 1. (**E**) Expression of four immune checkpoint molecules genes in the two clusters.

To identify the potential biological characteristics of the two TME clusters, differentially expressed genes (DEGs) were identified between TME Cluster 1 and Cluster 2. In TME Cluster 1, 37 genes were significantly upregulated and 778 genes were significantly downregulated when compared with the expression in Cluster 2 (Figure 2A; P < 0.05). DEG expression between the two clusters was visualized using a heatmap (Figure 2B). Kyoto Encyclopedia of Genes and Genomes (KEGG) enrichment analysis of the DEGs revealed enrichment of immune-related pathways, such as “leukocyte migration” and “leukocyte proliferation” in TME Cluster 2, which was supportive of the high immune cell infiltration patterns in this cluster (Figure 2C). In TME Cluster 1, significant upregulated genes were associated with development (Figure 2D), which were not immune-related. We further examined the expression of several immune checkpoint molecule genes between the two clusters, and found that four genes, *PD-1*, *PD-L1*, *CTLA-4*, and *TIM-3*, were significantly upregulated in TME Cluster 2 than in TME Cluster 1 (Figure 2E).

### Establishment and validation of TME signature

**Figure 3:**
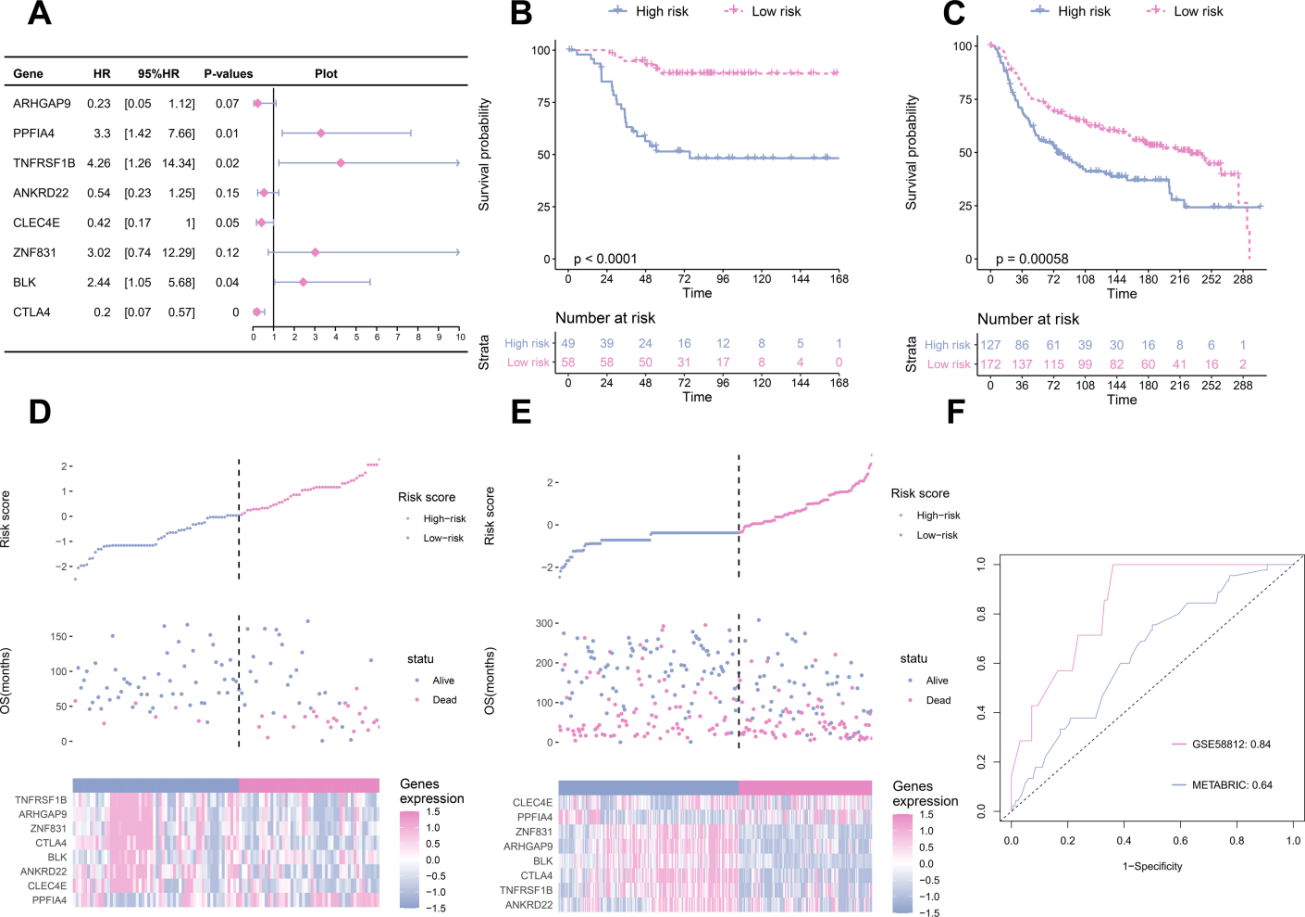
**TME signature is a promising marker of survival in TNBC patients.** (**A**) the HR and P-value from the eight genes in the prognostic model. (**B**) The survival curves for patients with high-risk and low-risk in the training set. (**C**) The survival curves for patients with high-risk and low-risk in the validation set. (**D**) Distribution of risk scores, survival profiles, and heat maps showing characteristic expressions of the low and high risky groups in the training set. Top panels indicate risk scores of patients. Middle panels depict survival status and survival time of patients distributed by risk score. Bottom panels display heatmap of expression for eight predictive factors distributed by risk score. (**E**) Distribution of risk scores, survival profiles, and heat maps showing characteristic expressions of low- and high-risk groups in the validation set. (**F**) Comparison of the predictive accuracy of the training set (GSE58812) and the validation set (METABRIC).

**Figure 4:**
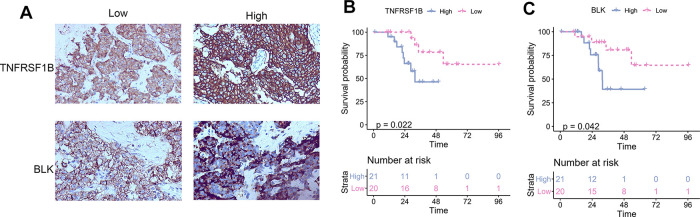
**The correlation between TNFRSF1B and BLK expression levels and TNBC prognosis.** (**A**) Representative immunohistochemical figure showed the high and low expression of TNFRSF1B and BLK. (**B**) The correlation between TNFRSF1B expression levels and TNBC prognosis. (**C**) The correlation between BLK expression levels and TNBC prognosis.

To establish a prognostic prediction model by TME-related characteristics, we applied univariate Cox regression to the survival data of the TNBC patients and expression profiles of 815 DEGs. Genes with P values < 0.1 in the univariate Cox regression were further subjected to multivariate Cox regression and stepwise regression [16, 17]. The final TME signature consisted of eight genes, *PPFIA4*, *TNFRSF1B*, *ARHGAP9*, *ZNF831*, *CTLA4*, *BLK*, *ANKRD22*, and *CLEC4E*. The hazard ratio (HR) and P values of the eight genes in the TME signature are shown in Figure 3A. Next, TME scores were obtained according to the TME signature, and the patients were categorized into high- and low-risk groups according to the median TME score. Survival analysis demonstrated that the TME scores exhibited strong power to distinguish good outcomes from poor in the TNBC patients in the training set (P < 0.001; Figure 3B). Patients at high-risk had significantly shorter survival compared to those at low-risk. To confirm our findings, we validated the TME signature in the validation set. Using the same model in the training set, we calculated the TME scores for each patient in the validation set, and evaluated the relationship between the TME score and their survival status. As shown in Figure 3C, patients at high-risk had significantly shorter survival compared to those at low-risk in the validation set. The distribution of the risk scores, survival statuses, and prognostic gene expression in the training and validation sets is shown in Figures 3D, 3E. To assess the prediction accuracy of the prognostic prediction model, receiver operating characteristic (ROC) curve was used. The ROC curve showed that the TME score demonstrated good discrimination in the training set with an area under the curve (AUC) of 0.84, and an acceptable discrimination in the validation set with an AUC of 0.64 (Figure 3F). Together, these results indicated that the prognostic prediction model based on the TME scores could be used to predict the survival of the TNBC patients. Since proteins seldom affect in isolation, it is important to know the interactions between the genes included in TME signature to identify the hub genes. We constructed a protein-protein interaction (PPI) network using STRING database. The PPI network comprised 8 nodes and 10 edges (Supplementary Figure 3). *TNFRSF1B* and *BLK* had the maximum neighboring genes, and were identified as the hub genes. We used immunohistochemistry (IHC) to analyze the correlation of *TNFRSF1B* and *BLK* expression with the prognosis of TNBC patients using an in-house paraffin embedded sample. Representative IHC figures are shown in Figure 4A. TNBC patients with high expression of either *TNFRSF1B* or *BLK* showed poorer prognosis than those with low expression of these hub genes (Figure 4B, 4C). These results were also consistent with the TME signature (HRs of *TNFRSF1B* and *BLK* were >1; Figure 3A), suggesting that *TNFRSF1B* and *BLK* were risk factors for poor prognosis in TNBC patients.

### Correlation of TME signature with different TME clusters

**Figure 5:**
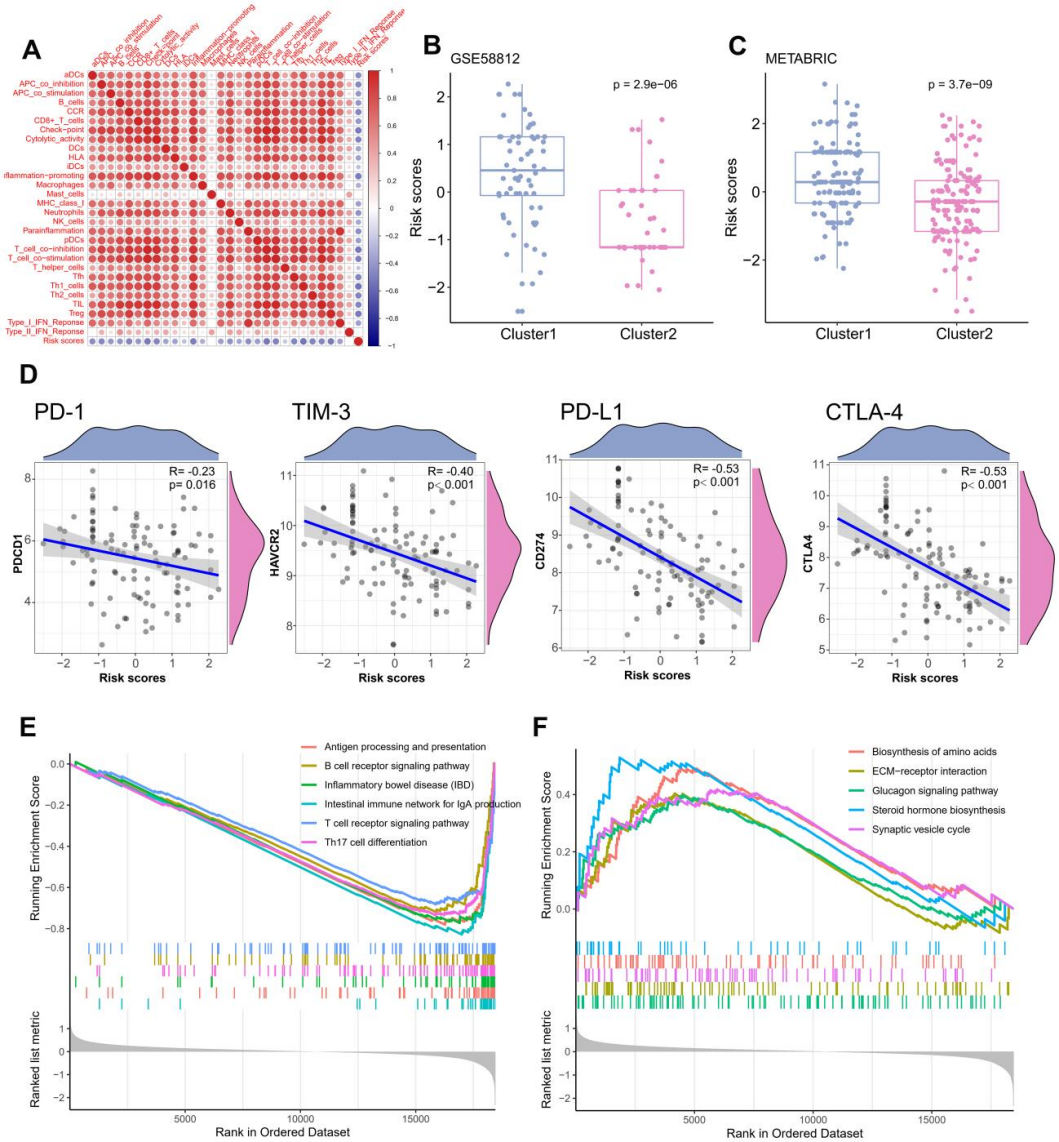
**Correlation of TME signature with different TME clusters.** (**A**) The correlation between TME signature and infiltrating immune cells. (**B**) Distribution of TME scores in different TME clusters of training set. (**C**) Distribution of TME scores in different TME clusters of the validation set. (**D**) The correlation between TME scores and immune checkpoint molecules. (**E**) Pathways enriched in low-risk group. (**F**) Pathways enriched in high-risk group.

**Figure 6:**
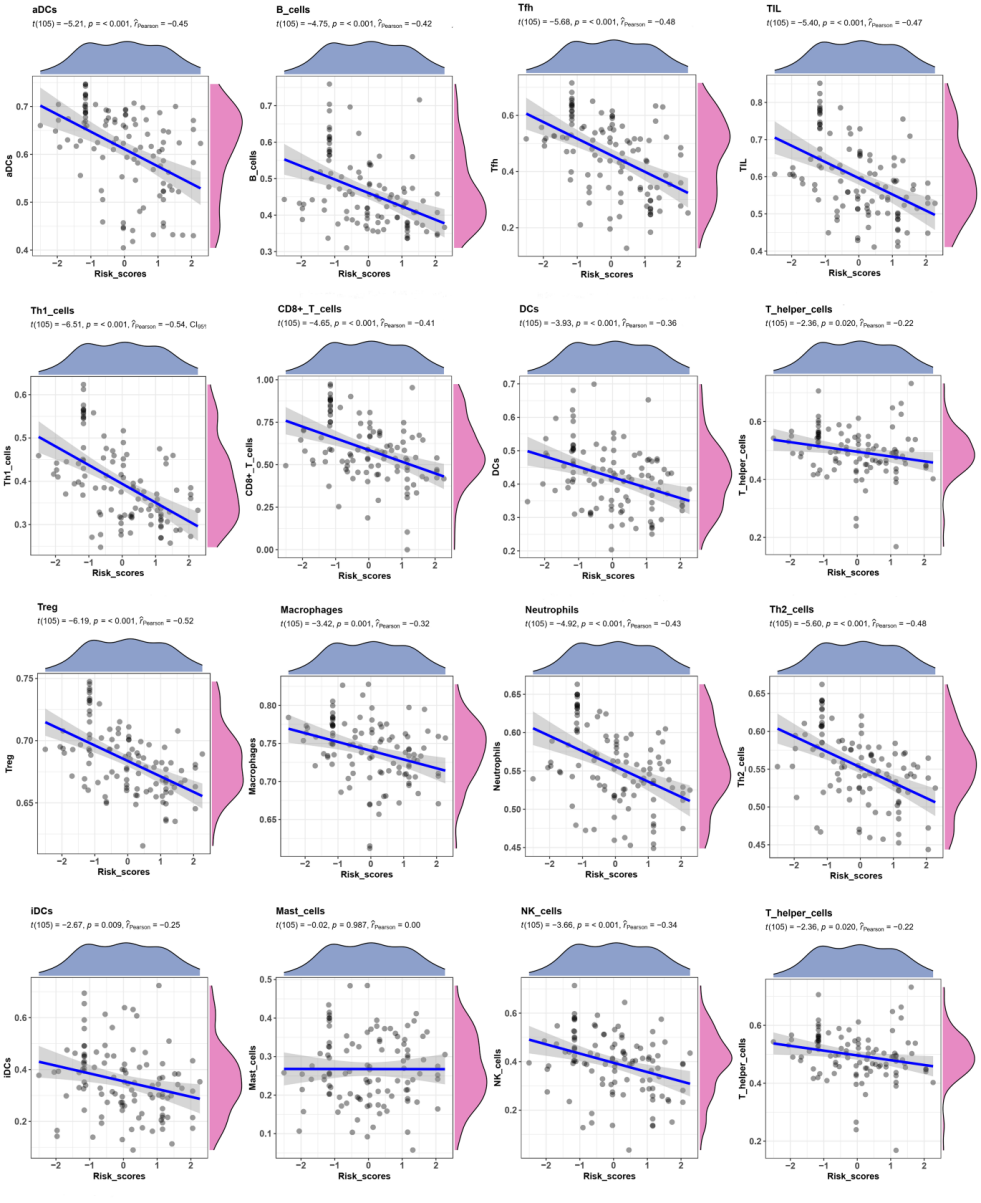
**Correlation between TME score and Tumor-infiltrating immune cells.** The correlation between TME scores with 16 kinds of tumor-infiltrating immune cells.

Relationship between the TME signature and TME clusters were evaluated by a correlation analysis. A TME cell network, depicting a comprehensive landscape of tumor-immune cell interactions with TME scores, was constructed. There was an almost all-round positive correlation between the abundance of the 29 immune-related terms. This phenomenon was most likely due to co-infiltration effects (Figure 5A). Interestingly, the TME scores were negatively correlated with all immune-related terms, indicating that the TME scores reflected the “cold” intratumoral microenvironment of TNBC. The association of TME scores with TNBC in different clusters was also analyzed, and the results showed that the high-infiltration group had a lower score than the low-infiltration group in the training set (Figure 5B). The same trend was also observed in the validation set (Figure 5C). Since the expression of immune checkpoint molecules, such as PD-1, were promising predictive factors for immune treatment response, we explored the association between immune checkpoint molecule expression and TME scores. Interestingly, TME scores were negatively correlated with all four immune checkpoint molecules (PD-1, PD-L1, CTLA-4, and TIM-3; P < 0.05; Figure 5D). To determine whether the patients with high- or low-risk scores were enriched for genes in previously defined biological pathways, GSEA analysis was performed. The results showed that in the low-risk score group, “intestinal immune network for IgA production,” “Th17 cell differentiation,” “B cell receptor signaling pathway,” “T cell receptor signaling pathway,” and “inflammatory bowel disease” were significantly upregulated (Figure 5E). In contrast, in the high-risk score group, “steroid hormone biosynthesis,” “synaptic vesicle cycle,” “biosynthesis of amino acids,” “ECM-receptor interaction,” and “glucagon signaling pathway” were the predominantly upregulated pathways (Figure 5F). Since the 29 immune-related terms contained 16 types of immune cells, we analyzed the association between the TME score and tumor-infiltrating immune cells. Th1 cells, CD8+ T cells, Treg cells, T cells, and 12 other immune-related cell types were shown in Figure 6. A negative correlation was found between the TME score and the tumor-infiltrating immune cells, indicating that the TME scores reflected the intrinsic characteristics of the TME.

### Correlation of TME signature with clinical parameters

**Figure 7:**
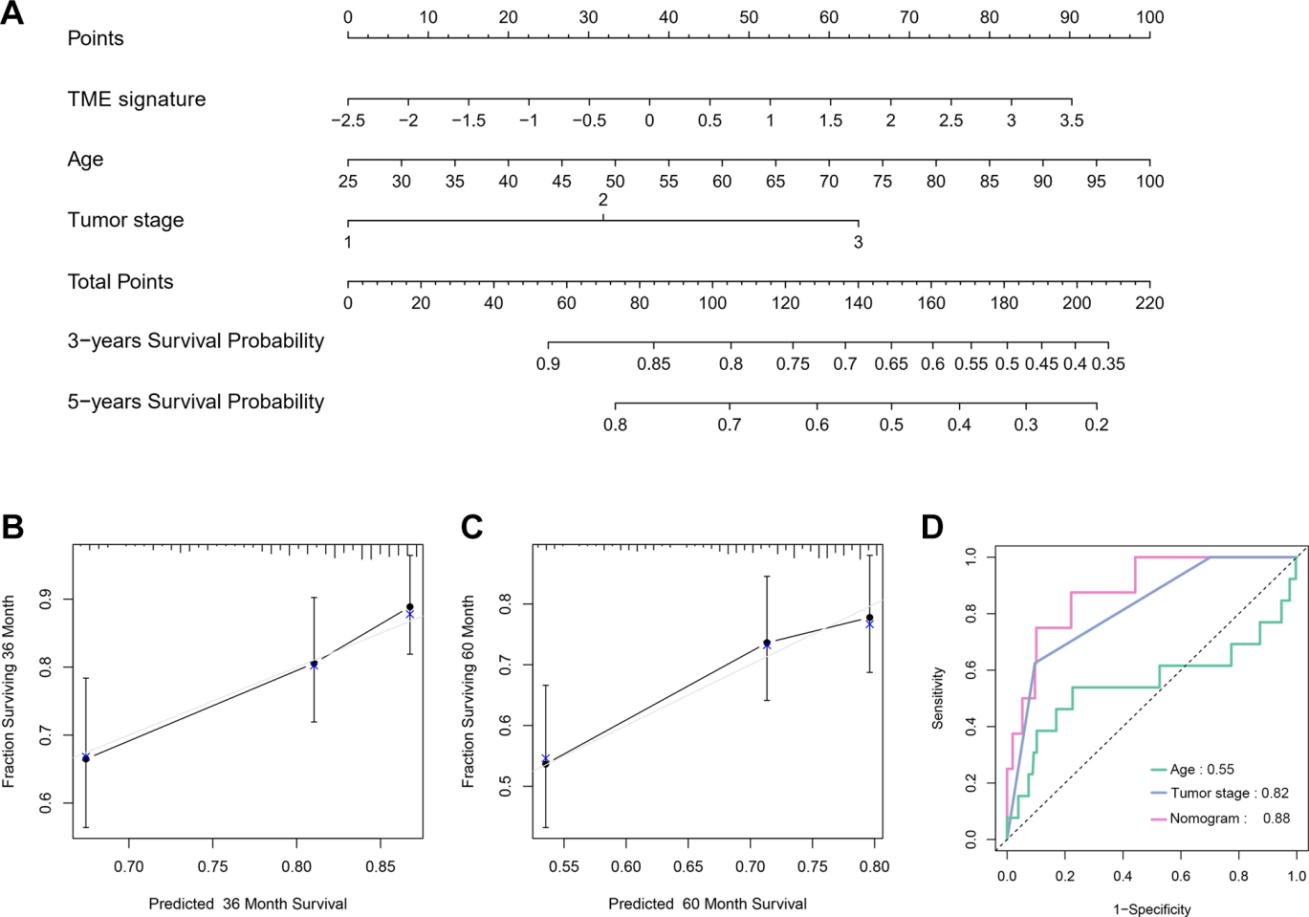
**Nomogram construction and evaluation.** (**A**) Nomogram for predicting 3- and 5-year survival probabilities of TNBC. Three points are allocated for age, tumor stage and TME signature. Draw a vertical straight line from the variable value to the axis labeled “Points”. Then calculate all variables’ points. The total points on the bottom scales that correspond to the 3- and 5-y survival were showed apparently. (**B, C**) Calibration curves for predicting 3-y (**B**) and 5-y (**C**) OS. Predicted survival produced by nomogram is plotted on the x-axis, and actual survival is plotted on the y-axis. Dashed lines represent an identical calibration model in which predicted OS approximate to actual OS. (**D**) Comparison of the predictive accuracy of nomogram, tumor stage and age.

We further investigated the relationship between the TME signature and clinical parameters. The patients were categorized into two groups according to the median of the TME scores. High TME scores were significantly associated with larger tumor sizes (P < 0.001; Supplementary Table 1). Univariate Cox analysis indicated that age, tumor stage, and TME signature were significantly associated with a poor prognosis of TNBC (all P < 0.05; Table 2). Multivariate Cox analysis indicated that age, tumor stage, and TME signature were independent prognostic factors for TNBC (Table 2). We constructed a nomogram based on three independent prognostic factors: age, tumor stage, and TME signature (Figure 7A) to obtain the estimated 3- and 5-year survival probabilities. To assess the calibration of the nomogram, we compared the predicted 3- and 5-year survival probabilities to the actual 3- and 5-year survival probabilities, and found that the calibration curve revealed good concordance between the predicted and observed probabilities (Figure 7B, 7C). These results indicated that the nomogram had proper calibration, and the ROC curve demonstrated that the nomogram had good discrimination with an AUC of 0.88, which was superior to that of the tumor stage (AUC = 0.82) and age (AUC = 0.55) (Figure 7D).

**Table 2:** Univariate and multivariate analyses of TME signature with common clinical parameters.

**Variable**	**Univariate analysis**			**Multivariate analysis**		
	**HR**	**95%CI**	**P-values**	**HR**	**95%CI**	**P-values**
Age	1.02	1.01-1.04	0.00011*	1.02	1.01-1.04	0.0066*
Cellularity	0.92	0.74-1.14	0.44			
Tumor stage	1.63	1.20-2.20	0.0016*	1.61	1.20-2.15	0.0014*
Grad	1.05	0.70-1.56	0.83			
TME signature	1.31	1.11-1.54	0.0012*	1.25	1.04-1.51	0.017*
Laterality	1.10	0.79-1.52	0.57			
Tumor size	1.45	0.93-2.25	0.10			

*P<0.05.

## DISCUSSION

In this study, the TNBC patients were categorized into two clusters: Cluster 1 and Cluster 2. Cluster 1 was associated with low immune infiltration and poor prognosis, while Cluster 2 was associated with high immune infiltration and good prognosis. Further, the DEGs in Cluster 2 included highly expressed genes that were mainly associated with immunity, and enriched in immune-related pathways. Furthermore, a prognostic prediction TME score method was developed based on the DEGs, and this score was negatively correlated with immune infiltration. Thus, our findings enhance the understanding of the tumor immune microenvironment in TNBC.

In recent years, an increasing number of studies have demonstrated that the TME, particularly the immune microenvironment, is associated with the development and progression of TNBC [18]. Lehmann et al and Burstein et al have reported that a subtype of TNBC displayed upregulated immunological responses, immune cell markers, and immune transcription factors, thereby implying the dysregulation of immune pathways in TNBC. Consequently, such studies have suggested that an immunotherapeutic approach may be a promising treatment strategy for TNBC patients [19].

In our study, Cluster 2 was associated with high immune infiltration, low TME score and good prognosis. The T cell immune response is a central event in antitumor immunity [20], with CD4+ (helper T cells, Th) and CD8+ (cytotoxic T cells, Tc) T cells secreting factors such as IFN-γ, TNF-α, and IL17 that have antitumor effects. Cluster 2 showed high expression of pDCs, iDCs, macrophages, B cells, highly permeable T cells, and cytotoxic T cells. This finding could explain the fact that Cluster 2 patients showed better OS and MFS than that of Cluster 1.

Some immune-related pathways were significantly enriched in Cluster 2 than in Cluster 1, which perhaps indicates a more active immune response in Cluster 2. The active immune microenvironment could further explain the better prognosis observed in Cluster 2. In addition, Cluster 2 appeared to coexist with higher levels of immune checkpoint molecules, such as PD-L1, PD-1, CTLA4, and TIM-3. Increased PD-L1 expression in immune cells of TME is accompanied by an increase in tumor-infiltrating lymphocytes and effector T cells [21, 22]. In addition, patients who are positive for PD-L1 protein on immune cells significantly benefit from PD-L1 blockade [23]. These findings suggest that higher expression of PD-L1 protein on immune cells is associated with a better response to immunotherapy and prognosis; thus, the patients in Cluster 2 might have been more sensitive to immunotherapy than those in Cluster 1. Similarly, the TME signature we constructed also indicated that high expression levels of PD-1, PD-L1, and CTLA-4 were correlated with a low TME score. Therefore, patients with a low TME score might benefit more from immunotherapy than patients with a high-risk score. However, the effectiveness of the classification of patients according to TME immune-related terms should be further assessed in large prospective clinical trials.

Next, we built a TME signature that included eight genes, *PPFIA4*, *TNFRSF1B*, *ARHGAP9*, *ZNF831*, *CTLA4*, *BLK*, *ANKRD22*, and *CLEC4E*. The protein encoded by *TNFRSF1B* is a member of the TNF receptor superfamily that mediates the recruitment of two antiapoptotic proteins, C-IAP1 and C-IAP2, with E3 ubiquitin ligase activity. With respect to *TNFRSF1B*, susceptibility to BC reduces with the rs1061622 GT genotype and G allele, but increases with the TA (rs1061622 T-rs1061624 A) haplotype, indicating that *TNFRSF1B* might affect BC risk [24]. GTPase activating proteins (RhoGAPs) play an important role in several aspects of tumor biology. In BC, high expression of *ARHGAP9* (gene encoding RhoGAPs) correlates with better relapse-free survival and OS, thereby suggesting that *ARHGAP9* may be a potential target for the precise treatment of BC [25]. Further, CTLA-4 is an efficient immunomodulatory molecule that downregulates T cell activation and suppresses the antitumor immune response. CTLA-4 is expressed in human BC cells and functions *in vitro* by influencing dendritic cell (DC) maturation and function. Blocking CTLA-4 restores DC maturation, promotes cytokine production, enhances DC antigen-presenting function, reverses Th1/CTLs response, and inhibits the biological activity of BC cells [26]. Furthermore, *BLK* encodes a non-receptor tyrosine kinase of Src family of proto-oncogenes, whose members promote the aggressiveness of BC cells, and enhance the antitumor effects of polyamine depletion upon inhibition [27]. Gene knockdown and overexpression analyses have shown that *ANKRD22* promotes tumor progression via increasing cell proliferation. In xenograft experiments, knockdown of *ANKRD22* or *in vivo* treatment with *ANKRD22* siRNA inhibits tumor growth [28]. CLEC4E (also called MINCLE) drives the maturation of antigen-presenting cells and shifts T cells toward effector Th1 and Th17 cell subtypes to form antigen-specific triggers. In addition, in the TME, MINCLE induces immune suppression and cancer progression, which are achieved by macrophages [29]. Thus, these key genes may be new therapeutic targets for TNBC and further mechanistic research is required to elucidate their role in TME of TNBC.

Although this study has demonstrated valuable insights regarding TNBC, its limitations should be acknowledged. Firstly, this is a retrospective study; therefore, the robustness of the predictive value of the gene signature should be further validated. Secondly, downstream functional studies are required to further elucidate the biological function of the prognostic gene signature identified in this study. Finally, although tissue microarray (TMA) enables efficient gene expression profiling of large number of samples, it may only reflect averaged cell components and immune activation in TME. Thus, TMA has limited ability to capture the heterogeneity of infiltrating immune cells, immune responses, and tumor cells. As a result, the effectiveness of our model requires further investigations.

## CONCLUSIONS

In this study, we analyzed the TME clusters of TNBC and their relationship with clinical characteristics. A TME signature for prognostic prediction of TNBC was developed and validated, and patients were categorized into high- and low-risk groups according to their TME scores. The prognosis of the two groups was found to be significantly different. Further, we identified a cluster of TNBC patients with high immune infiltration, who may show a high response rate to immunotherapy. We believe that this study improves the understanding of the immune microenvironment; thus, the analysis of immune infiltration patterns of TME may provide new insights for immunotherapy in TNBC cases and guidance for the development of novel drug combination strategies.

## MATERIALS AND METHODS

### Clinical specimens

A total of 41 specimens of TNBC tissues were obtained from patients at the Guangxi Medical University Cancer Hospital, People’s Republic of China between 2011 and 2019. TNBC was pathologically confirmed in all patients who were not on chemotherapy or radiotherapy before the collection of the tissues. Written informed consent was obtained from all patients, and the study was approved by the Ethics and Human Subject Committee of Guangxi Medical University Cancer Hospital. All experiments and methods were performed according to relevant guidelines and regulations formulated by the Guangxi Medical University Cancer Hospital.

### Data acquisition and processing

We retrieved two transcriptomic expression datasets of BC cohorts from public databases: a microarray dataset from Gene Expression Omnibus (GEO; https://www. ncbi.nlm.nih.gov/geo/) database with accession number GSE58812 [30] and a dataset of clinical and mRNA expression from the Molecular Taxonomy of Breast Cancer International Consortium (METABRIC; cBioportal of Cancer Genomics (https://www.cbioportal.org/) [31]. For GSE58812, the probes in the microarray data were annotated according to Affymetrix Human Genome U133 Plus 2.0, batch effects caused by different studies were removed by “ComBat” (empirical Bayes method) from the “sva” package [32, 33], and background noise was cancelled via data normalization using the “limma” package [34]. For the METABRIC cohort, Illumina HT-12v3 platform was used to measure mRNA expression. METABRIC data was formerly median-centered and log-transformed. GEO and METABRIC datasets were utilized for model development and validation, respectively.

### IHC analysis

All tissue specimens obtained from the patients were fixed in 10 % neutral formalin and then embedded using paraffin. The embedded specimens were cut into 5 μM serial sections just before the staining. IHC was performed using SPlink Detection Kits SP-9000 (ZSGB-BIO, China) according to the manufacturer’s instructions. The sections were processed by deparaffinization, rehydration, and blocking of endogenous peroxidase activity, and then incubated with primary antibodies (BLK, 1:200; ABCEPTA and TNFRSF1B, 1:200, ALS17735, ABCEPTA) overnight at 4° C. Next, the sections were incubated with a secondary antibody, i.e., a biotinylated donkey anti-goat IgG and horseradish peroxidase-labeled streptomycin working solution, and then treated with diaminobenzidine hydrochloride to visualize the immunoreactivity. The immunohistochemical scoring was performed independently by two authors who were blinded to the clinicopathological parameters. The score was composed of two parts. One was the extent of staining (percentage of positive tumor cells: 0 % = 0; 1–25 % = 1; 26–50 % = 2; 51–75 % = 3; and 75-100 % = 4), and the other was the intensity of staining (no staining = 0; weak staining = 1; moderate staining = 2; and strong staining = 3). The total scores that ranged from 0 to 4 were considered “low expression,” while those that ranged from 5 to 7 were considered “high expression.”

### TME cell abundance calculation

To investigate the TME immune cell abundance in TNBC, we methodically examined published studies, adopted the gene signatures proposed by Bindea et al [35], and obtained the marker gene sets for different immune cell types. Gene signatures signifying microenvironment cell subsets of both adaptive and innate immunity were also obtained. TNBC with qualitatively different immune cell infiltration patterns were grouped by hierarchical agglomerative clustering, depending upon Ward's linkage and Euclidean distance. ssGSEA, from the R package “GSVA,” was applied to identify the enrichment scores of each immune-related term to quantify the infiltration levels of different immune cell types in TME [32]. In addition, TIMER2.0 and CIBERSORTx, were employed to validate the TME cell abundance calculated by ssGSEA using default parameters. TIMER2.0 integrates multiple state-of-the-art algorithms for estimating six types of tumor infiltrating immune cells [36]. Meanwhile, CIBERSORTx is a deconvolution algorithm based on support vector regression, which uses a set of reference gene expression values corresponding to a minimal representation for each cell type to infer the cell type proportions in data from bulk tumor samples with mixed cell types [37]. CIBERSORTx can sensitively and specifically discriminate between 22 human immune cell phenotypes.

### Unsupervised cluster analysis

We identified characteristic expression patterns based on the infiltration of immune cells by using unsupervised clustering analysis based on Euclidean distance and Ward's linkage. The patients were categorized for further analysis. We established the best number of “TMEclusters” based on the percentage of variance of the data by applying the “ConsensusClusterPlus” package with 1000 repeats [38]. We categorized the patients into two groups based on the infiltrating degrees of the immune cells. Finally, we calculated the correlation matrix using Spearman's test and generated images using R package “ggplot2.”

### Identification of DEGs and construction of PPI networks

In order to identify the DEGs between the two distinct TME clusters, we used the R package “limma” [39]. P values were adjusted by Benjamini-Hochberg correction, and DEGs with adjusted P value < 0.05 and |log2 (fold change)| > 1 were retained. PPI networks were constructed using STRING (https://string-db.org/) with confidence of interaction set at 0.15 [40]. The networks were visualized by Cytoscape version 3.6.0 [41].

### Enrichment analysis

DEGs were subjected to KEGG and Gene Ontology (GO) pathway enrichment analyses, and only terms with adjusted P < 0.05 were considered significant. The “GOSemSim” R package was used to assess the similarity between the significant GO terms of the two groups by referring to the annotation data GO.db. Similar GO terms between two groups were graphically represented by a heatmap and tree diagram [42]. The median TME signature was used as a cutoff value to classify the patients into high- and low-risk groups. Then, gene set enrichment analysis (GSEA) was performed to test whether the genes in the high- or low-risk groups were enriched in the predefined KEGG gene sets. After 1000 permutations, the gene sets with P < 0.05 and false discovery rate < 0.05 were considered significant.

### TME signature construction

Unsupervised clustering was used to categorize the patients into two TME clusters (Cluster 1 and Cluster 2). Based on the DEGs, a univariate Cox regression analysis was performed to identify the prognostic DEGs by applying the “survival” R package. To build the TME signature, DEGs with P value < 0.1 in the univariate Cox regression were considered the prognostic genes. Next, we used a stepwise multivariate Cox regression analysis to screen for key prognostic genes. Then, we computed the TME signature for each patient using the following formula:

TME signature = β1 × exprG1 + β2 × exprG2 + ... βn × exprG

where exprG is the expression level of the vital prognostic genes and β is the regression coefficient computed from the multivariate Cox regression model. The GSE58812 cohort was used as the training set to develop the TME signature, and the METABRIC cohort was used as the validation set to confirm the stability of the TME signature. A forecast value was calculated for the patients in the validation set according to the risk model that was made in the training set. The ROC curve and AUC were used to calculate the predictive risk model’s discrimination ability.

### Construction of the nomogram

Nomograms have recently attracted increased attention as a user-friendly tool for predicting prognosis with strong clinical utility [43, 44]. TME signature and clinical parameters were subjected to univariate Cox proportional hazards analyses. Features with P values < 0.05 were subjected to multivariate Cox proportional hazards analysis. Features with P values < 0.05 after multivariate analysis were incorporated into nomograms that were constructed to predict the 3- and 5-year survival rates [45]. The nomogram was based on three independent prognostic factors: age, tumor stage, and TME signature. Each factor corresponded to a specific point by drawing a line straight upwards to the points axis. The sum of the three factor points was defined as total points. By drawing a perpendicular line from the total point axis to the two-outcome axes, the estimated 3- and 5-year survival probabilities were obtained. The observed 3- and 5-year survival rates were compared with the predicted 3- and 5-year survival rates to further verify the predictive performance of the nomogram. We assessed the goodness-of-fit of the nomogram using calibration plots [46].

### Statistical analysis

For comparison between two groups, non-parametric Mann-Whitney U test and parametric unpaired Student’s *t*-test were used. For comparison between more than two groups, non-parametric Kruskal-Wallis test and parametric one-way analysis of variance (ANOVA) test were used. Spearman and distance analysis were used to perform correlation analysis. The survival curves concerning the prognostic analysis were prepared by Kaplan-Meier method, and log-rank tests were employed to identify the significance of the survival differences. We used the "LR forward" stepwise approach and Cox proportional risk model for the univariate and multivariate analyses. To assess the accuracy of survival prediction by the prognostic model, we used a time-related ROC analysis. All statistical analyses were performed in R version 3.5.0. Two-sided P value < 0.05 was regarded as significant.

### Ethics approval and consent to participate

This study was approved by the Ethics and Human Subject Committee of Guangxi Medical University Cancer Hospital. All patients provided written informed consent.

### Availability of data and materials

The dataset supporting the conclusions of this article is included within the article.

## Supplementary Material

Supplementary Figures

Supplementary Table 1
