# Supplementary material for: Tumor microenvironment characterization in triple-negative breast cancer identifies prognostic gene signature
Source: Aging (Albany NY). 2021 Feb 1;13(4):5485–505. doi: 10.18632/aging.202478 (PMC7950290; doi:10.18632/aging.202478)
Supplement: Supplementary Figures [file aging-13-202478-s001.pdf]

SUPPLEMENTARY FIGURES

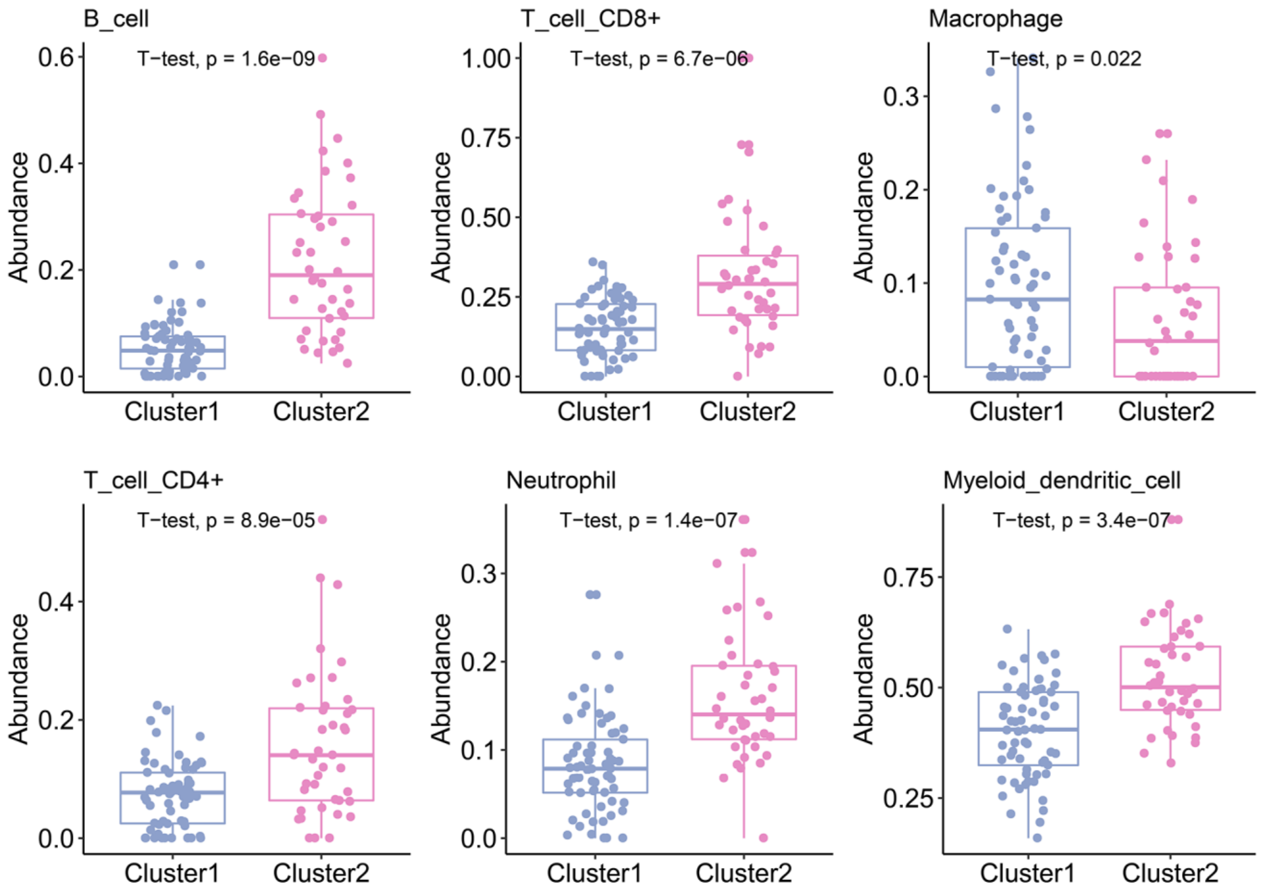

Supplementary Figure 1. The abundance of tumor infiltrating immune cells in the Cluster 1 and 2 estimated by TIMER2.0. TIMER2.0 estimated 6 kinds of tumor infiltrating immune cells.

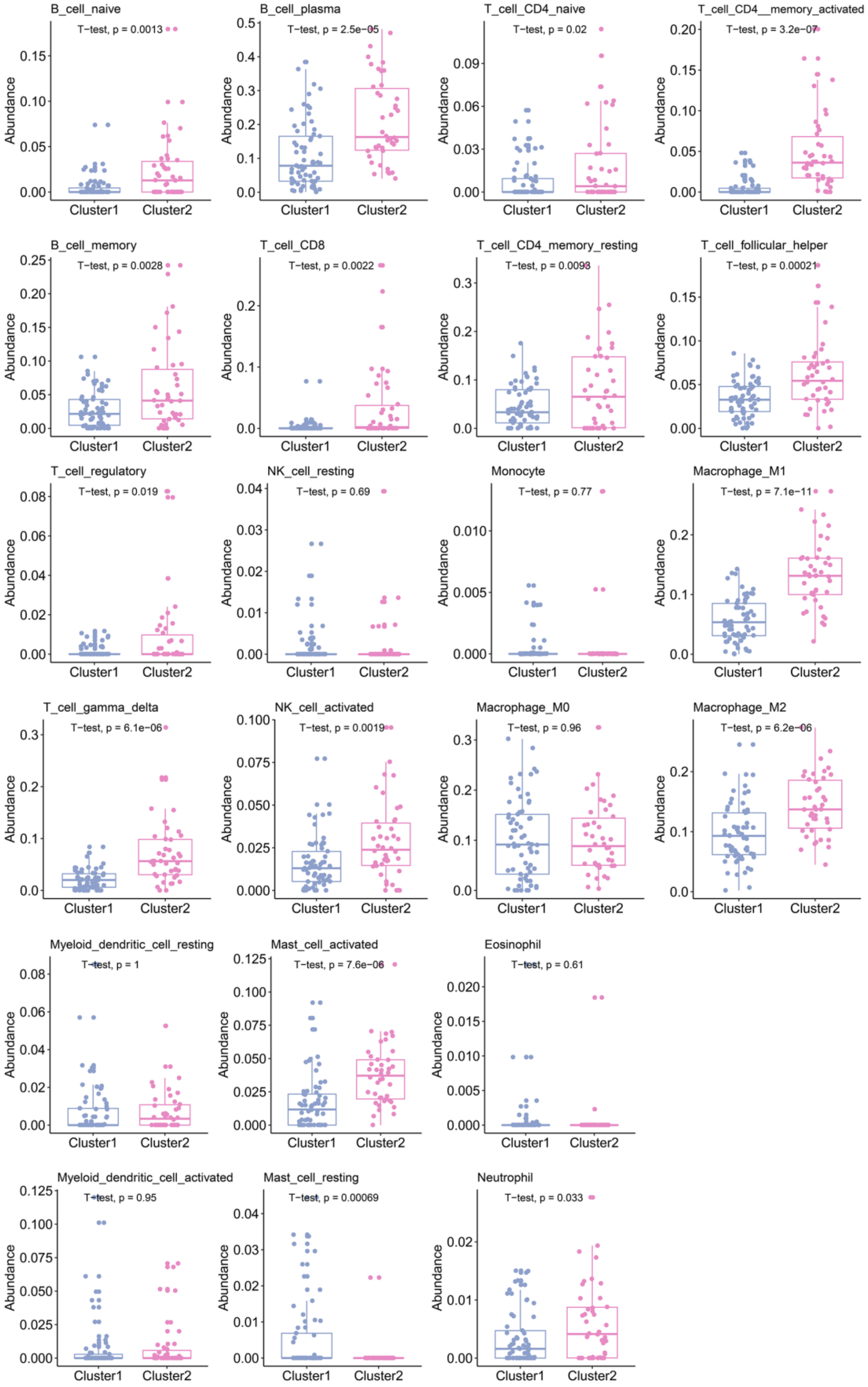

Supplementary Figure 2. The abundance of tumor infiltrating immune cells in the Cluster1 and 2 estimated by CIBERSORTx. CIBERSORTx estimated 22 kinds of tumor infiltrating immune cells.

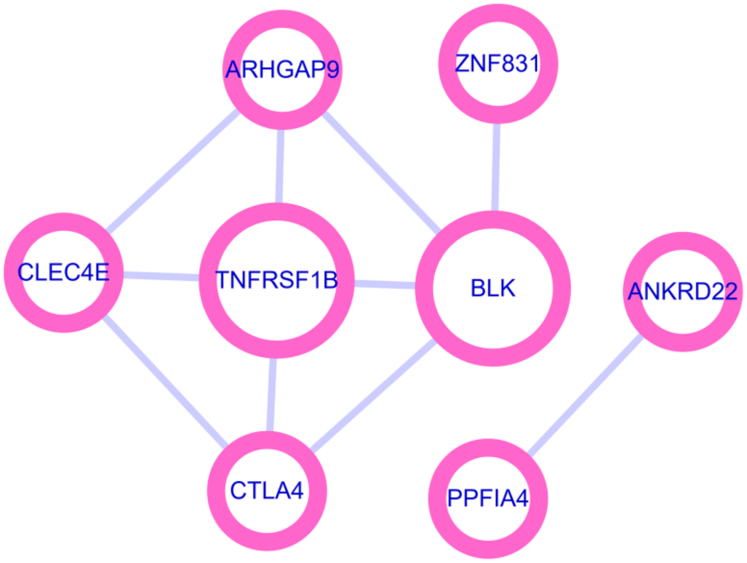

Supplementary Figure 3. Protein-protein interaction network of 8 genes in the TME signature. TNFRSF1B and BLK with the most neighbor genes were identified as hub genes.
