# Supplementary material for: Tumor microenvironment characterization in triple-negative breast cancer identifies prognostic gene signature
Source: Aging (Albany NY). 2021 Feb 1;13(4):5485–505. doi: 10.18632/aging.202478 (PMC7950290; doi:10.18632/aging.202478)
Supplement: Supplementary Table 1 [file aging-13-202478-s002.pdf]

SUPPLEMENTARY TABLE

Supplementary Table 1. Clinical characteristics of the TNBC patients with different TME scores.

Characteristics	Low risk group	High risk group	P value
Age (mean (SD))	54.52(13.01)	57.16(14.58)	0.100
NPI (mean (SD))	4.62(0.92)	4.55(0.99)	0.524
Cellularity (%)			0.412
High	88(51.2)	76(59.8)	
Low	21(12.2)	15(11.8)	
Moderate	56(32.6)	34(26.8)	
NA	7(4.1)	2(1.6)	
Chemotherapy (%)			0.510
Yes	87(50.6)	70(55.1)	
No	85(49.4)	57(44.9)	
Inferred Menopausal State (%)			0.227
Pre	67(39.0)	40(31.5)	
Post	105(61.0)	87(68.5)	
Laterality (%)			0.694
Left	79(45.9)	60(47.2)	
Right	85(49.4)	57(44.9)	
NA	8(4.7)	10(7.9)	
Grade (%)			0.690
1	1(0.6)	2(1.6)	
2	21(12.2)	15(11.8)	
3	149(86.6)	108(85.0)	
NA	1(0.6)	2(1.6)	
Tumor size (mean (SD))	23.88(10.28)	32.89(24.42)	<0.001*
Tumor stage (%)			0.233
1	37(21.5)	25(19.7)	
2	81(47.1)	49(38.6)	
3	11(6.4)	14(11.0)	
NA	43(25.0)	39(30.7)	
Positive lymph nodes (%)			0.900
>5	21(12.2)	17(13.4)	
≤5	151(87.8)	110(86.6)	

NPI: Nottingham prognostic index.
* P<0.05.
